# Single- and Multilayered Perovskite Thin Films for Photovoltaic Applications

**DOI:** 10.3390/nano12183208

**Published:** 2022-09-15

**Authors:** Nawishta Jabeen, Anum Zaidi, Ahmad Hussain, Najam Ul Hassan, Jazib Ali, Fahim Ahmed, Muhammad Usman Khan, Nimra Iqbal, Tarek A. Seaf Elnasr, Mohamed H. Helal

**Affiliations:** 1 Department of Physics, Fatima Jinnah Women University, Rawalpindi 46000, Pakistan; 2 Department of Physics, Sargodha Campus, The University of Lahore, Sargodha 40100, Pakistan; 3 Department of Physics, Division of Science and Technology, University of Education, Lahore 54000, Pakistan; 4 Center for Hybrid and Organic Solar Energy (CHOSE), University of Rome Tor Vergata, 00133 Rome, Italy; 5 National Key Laboratory of Tunable Laser Technology, Institute of Optoelectronics, Department of Electronics Science and Technology, Harbin Institute of Technology, Harbin 150080, China; 6 Department of Chemistry, College of Science, Jouf University, Sakaka P.O. Box 2014, Aljouf, Saudi Arabia; 7 Department of Chemistry, Faculty of Arts and Science, Northern Border University, Rafha P.O. Box 1321, Northern Borders Region, Saudi Arabia

**Keywords:** perovskite, thin films, spin coating, methylammonium lead iodide bromide, ferroelectric, IV measurements, FTIR

## Abstract

Organic–inorganic lead halide perovskites materials have emerged as an innovative candidate in the development of optoelectronic and photovoltaic devices, due to their appealing electrical and optical properties. Herein, mix halide single-layer (~95 nm) and multilayer (average layer ~87 nm) CH_3_NH_3_PbIBr_2_ thinfilms were grown by a one-step spin coating method. In this study, both films maintained their perovskite structure along with the appearance of a pseudo-cubic phase of (200) at 30.16°. Single-layer and multilayer CH_3_NH_3_PbIBr_2_ thinfilms displayed leaky ferroelectric behavior, and multilayered thinfilm showed a leakage current of ~5.06 × 10^−6^ A and resistivity of ~1.60 × 10^6^ Ω.cm for the applied electric field of 50 kV/cm. However, optical analysis revealed that the absorption peak of multilayered perovskite is sharper than a single layer in the visible region rather than infrared (IR) and near-infrared region (NIR). The band gap of the thinfilms was measured by Tauc plot, giving the values of 2.07 eV and 1.81 eV for single-layer and multilayer thinfilms, respectively. The structural analysis has also been performed by Fourier transform infrared spectroscopy (FTIR). Moreover, the fabricated CH_3_NH_3_PbIBr_2_ as an absorber layer for photoelectric cell demonstrated a power conversion efficiency of 7.87% and fill factor of 72%. Reported electrical, optical and photoelectric efficiency-based results suggest that engineered samples are suitable candidates for utilization in optoelectronic and photovoltaic devices.

## 1. Introduction

Recently, optoelectronic devices have proven to be a special class of devices in research, used to generate light by electric charge and work in a way comparable to LASER and light emitting diodes (LED), or an electric current is generated by light to optimize solar cells and optoelectronic devices [1]. Optoelectronic devices can further be divided into light-generating and light-sensing devices which are their core features. In the past decades, two-dimensional materials, such as transition metal dichalcogenides, boron nitride, group-III and group-IV metal chalcogenides, black phosphorus, germanene and related composites/thinfilms/single crystals/ceramics/heterostructures, were engineered or fabricated to show extraordinary physical and chemical character for such devices [2]. In the present era of technology, rapid development in the efficiency of optoelectronic and photovoltaic devices has been observed, and to serve this purpose, the development of new-type of materials is required. Hybrid organic–inorganic perovskite photovoltaic cells display high efficiency above ~22%, which is due to the large carrier diffusion lengths, high absorption coefficients and high carrier mobility of perovskite absorber layers [3]. The structure of photovoltaic cells has grabbed the attention of research scholars, for such purposes as lead halide perovskites thinfilms deposited on different types of substrate, which has gained importance [4,5]. The consumption of thinfilms in efficient photovoltaic cells is proving to be the best energy source.

Lead halide perovskites (CH_3_NH_3_PbX_3_; X = Cl, Br and I) have provided a significant interest for researchers to explore their electronic and optical properties for optical devices. It is reported that these lead halide perovskites display high charge carrier mobility, a large absorption coefficient over a broad-spectrum range and long carrier diffusion length [6,7]. Sariful et al. reported that CH_3_NH_3_PbBr_3_ possesses a cubic structure with space group *Pm3m* crystal symmetry, while CH_3_NH_3_PbI_3_ possesses a tetragonal phase with *I4/mcm* space group [8]. Ryung et al. reported that the mixed halide perovskite single crystals (Br/I, 2:1) show the pseudo-cubic phase at room temperature [9]. Later, several research reports demonstrated that solid state CH_3_NH_3_PbI_3_ perovskite solar cells exhibited ~10–11% photo-electron conversion efficiency (PCE) with considerably better stability [10]. Better crystalline quality, high charge carrier mobility, carrier diffusion length, and absorption coefficient of the materials can play a vital role towards employment in efficient devices. These factors can be improved by selecting appropriate substrates and introducing laser-assisted synthesis protocols [11,12]. Afterwards, lead halide-based perovskite solar cells gained attention in terms of their photo-electron conversion efficiency, after incredible research reports on perovskite thinfilms deposition and interface engineering.

CH_3_NH_3_PbBr_3_ is another famous lead halide perovskite, which shows strong character for electronic and optoelectronic devices. Heo et al. deposited dense CH_3_NH_3_PbBr_3_ perovskite thinfilms on a TiO_2_/FTO substrate, which exhibited high device efficiency of ~7.3% [11]. Huifang et al. fabricated perovskite CH_3_NH_3_PbBr_3_ thinfilms following the vapor assisted solution method and revealed no structural phase transformation under the heating process of 10–300 K [12]. Jyoti et al. reported that CH_3_NH_3_PbBr_3_ thinfilm exhibited the ideality factor of ~2.37 in the dark and ~1.79 under illumination [13]. Wu et al. reported a Au-CH_3_NH_3_PbBr_3_ hybrid structured thinfilm, which demonstrated angular sensitivity of the fabricated thinfilm’s guided-wave surface plasmon resonance, as the biosensor is much higher (278.5°/RIU), ~110.2% greater than the conventional Au-based surface plasmon resonance biosensor [14]. For the CH_3_NH_3_PbX_3_ (X = Cl, I and Br) halide perovskites, cation, halide and metal composition all can play vital roles to modify the properties of the final material to be utilized in optoelectronic devices. Similarly, mixed cation perovskites can exhibit promising behavior to enhance both the photo-electron conversion efficiency and stability. Recently, dual halide perovskites such as CH_3_NH_3_Pb(Cl_x_Br_3-x_) or CH_3_NH_3_Pb(Cl_X_I_3-X_) were reported to be utilized in photo-detectors, solar cells, light emitting diodes and tunable lasers over a UV-NIR range [15]. Crystalline CH_3_NH_3_PbI_3–x_Cl_x_ thinfilms fabricated by solution process have shown the ability to convert 70% of absorbed light into emitted light [16]. Similarly, a CH_3_NH_3_PbBr_3–x_Cl_x_ semiconductor was tuned from 2.42 to 3.16 eV to construct a light-emitting diode [17]. There exist several reports on lead halide-based perovskite single crystals, composites, and single-layer thinfilms to be utilized in photovoltaic and optoelectric devices. With the growing need of energy reservation, there is a need to search for alternative directions to explore these existing materials. Multilayered lead-halide perovskite thinfilms can produce significant interest for researchers to investigate their electrical and optical properties to be utilized in such devices. In this article a comparative study is presented between dual lead halide-based perovskite single-layer and multilayered CH_3_NH_3_PbIBr_2_ thinfilms for the devices. However, there is still a need to perform more research to bring out the best properties from this approach. Moreover, the employment of perovskite CH_3_NH_3_PbIBr_2_ as an absorber layer for a photoelectric cell with 6.02% efficiency is still the highest in an inverted planar with CH_3_NH_3_PbIBr_2_ structure, and is also comparable with a regular planar structure in PCE.

In this study, lead dual-halide perovskite (CH_3_NH_3_PbIBr_2_) single- and multilayered thinfilms were fabricated to observe the performance application of the material in the photovoltaic cell. CH_3_NH_3_PbIBr_2_ as an absorber layer was employed for photoelectric cells to observe the power conversion efficiency. The achieved results demonstrated the ability of the engineered material for the utilization in photovoltaic and optoelectronic devices.

## 2. Experimental Section

### 2.1. Preparation of CH_3_NH_3_PbIBr_2_ Solution

Lead dual-halide perovskite (CH_3_NH_3_PbIBr_2_) single- and multilayered thinfilms were grown by one step solution method. High purity lead (II) bromide (PbBr_2_ with 98% purity), methyl-ammonium iodide (CH_3_NH_3_I with 99.8% purity), dimethyl-sulfoxide (DMS with 99.5% purity) and N,N-dimethyl-formamide (DMF with 99.8% purity) were purchased from Sigma Aldrich (St. Louis, MO, USA). All salts and solvents were used as received without any extra refinement. The 0.5 mL and 1.5 mL solvents of DMF and DMSO, respectively, were prepared using micro pipette to make the combination of (1:3) in the viol. After that, 159 g/mol of CH_3_NH_3_I was added in the 2 mL of solvents and the whole solution was stirred for 15 min at 50 °C by using magnetic stirrer. Finally, 367 g/mol of PbBr_2_ was added and then again the whole solution was stirred for 5 min at 70 °C using a magnetic stirrer. In this way, the dual-halide perovskite CH_3_NH_3_PbIBr_2_ solution was prepared.

### 2.2. Preparation of CH_3_NH_3_PbIBr_2_ Thinfilms

For the growth of CH_3_NH_3_PbIBr_2_ multilayered thinfilms, one-step spin coating technique was employed at the speed of 500 rpm for 10 sec and then at 3500 rpm for 40 sec. Prior to the fabrication of thin films, glass substrates (0.02 × 0.02 cm^2^) were sonicated with liquid detergent to remove dust particles from glass substrates for 15 min at 60 °C, then rinsed with DI water, agitated in acetone for 15 min to remove impurity particles, then the substrates were placed in isopropyl (IPA) to remove any remaining waste for 15 min in an ultrasonic bath and dried in hot air. For the measurements of electrical properties, silver (Ag) layer with thickness ~50–60 nm nm was deposited on the glass substrate (as bottom electrode) using an HR Vacuum Chamber under pressure of 10^−5^ Torr with a deposition rate 3–5 Å/s. After deposition of the silver layer, lead dual halide perovskite CH_3_NH_3_PbIBr_2_ thinfilm layer was grown by using one-step spin coating technique. The thinfilm was dried at 70 °C using hot plate. In a similar way (mentioned above), a Ag layer was deposited on the first perovskite CH_3_NH_3_PbIBr_2_ layer, followed by the growth of second lead dual halide perovskite CH_3_NH_3_PbIBr_2_. Three CH_3_NH_3_PbIBr_2_ layers alternative to the Ag layer were grown, and finally a Ag layer was deposited on the top of the thinfilm which will work as top electrode for electrical measurements.

Single-layered CH_3_NH_3_PbIBr_2_ thinfilm was grown by using a one-step spin coating technique (500 rpm for 10 sec and then at 3500 rpm for 40 sec). Initially, the Ag (bottom electrode) of thickness ~60 nm was deposited on the glass substrate (0.02 × 0.02 cm^2^) using HR Vacuum Chamber under pressure of 10^−5^ Torr with a deposition rate of 3–5 Å/s, followed by the growing of a single-layer CH_3_NH_3_PbIBr_2_ film (same CH_3_NH_3_PbIBr_2_ solution for single- and multilayered thinfilms). The thinfilm was dried at 70 °C using hot plate. Finally, using the same conditions, a top Ag electrode was deposited on the film.

### 2.3. Device Fabrications

For the fabrication of a photovoltaic device, commercially available indium tin oxide (ITO)-coated glass substrate with an active area of 0.04 cm^2^ was taken. These ITOs substrates were washed by ultra-sonication with detergent soap, DI water, isopropanol, Aceton and DI water, respectively, and each sonication step was 15 min long. These washed substrates were then dried inside a thermal dryer for a whole night. For the device fabrication, these washed substrates were put under UV ozone treatment for 15 min before transferring to the nitrogen-filled glove box. A PTAA solution was prepared by dissolving 2 mg/mL in Chlorobenzene which was stirred for a whole night at 60 °C and spin coated at 5000 rpm for 30 s on top of the ITO substrate. To obtain the CH_3_NH_3_PbIBr_2_ precursor solution, PbI:CH_3_NH_3_Br_2_ (1:2 M) was dissolved in a 7:3 *v*:*v* DMF:DMSO solvent and stirred overnight at 60 °C. This perovskite precursor solution was spin-coated in two steps at 1000 rpm for 10 s and 5000 rpm for 20 s, respectively. During the second step, Chlorobenzene (150 µL) was dripped out onto the spinning substrate for 10 s at the end of the program. The thinfilms were then annealed at 125 °C for 15 min. The PCBM solution was prepared by dissolving 20 mg of PC_60_BM into Chlorobenzene, which was then stirred for whole night at 60 °C and spin coated on top of the perovskite layer at 2000 rpm for 30 s. Finally, a 100 nm Aluminum (Al) electrode was deposited through thermal metal evaporation.

### 2.4. Characterization

X-ray diffraction was employed to monitor the structure of perovskite film using XRD; DX-2700 via Cu-Kα radiation (λ = 1.5416 Å). Scanning electron microscopy (FE-SEM, FEI Quanta 200, Hillsboro, OR, USA) was employed to examine the morphology of the perovskite thinfilms. The ferroelectric properties (*P-E* loops) were tested by a ferroelectric tester (aixACC TF Analyser 1000, aixACCT Systems GmbH, Aachen, Germany).

The electrical properties of the CH_3_NH_3_PbIBr_2_ single-layer and multilayered thinfilms were studied using a two-point probe source meter technique (KEITHLEY Instrument 2420 Model, Leeds, UK). Optical absorption and transmittance spectra of CH_3_NH_3_PbIBr_2_ (single-layered and multilayered) thinfilms were recorded using single beam Shimadzu UV-Visible Spectrometer 1900i (Kyoto, Japan).

## 3. Results and Discussion

**Figure 1:**
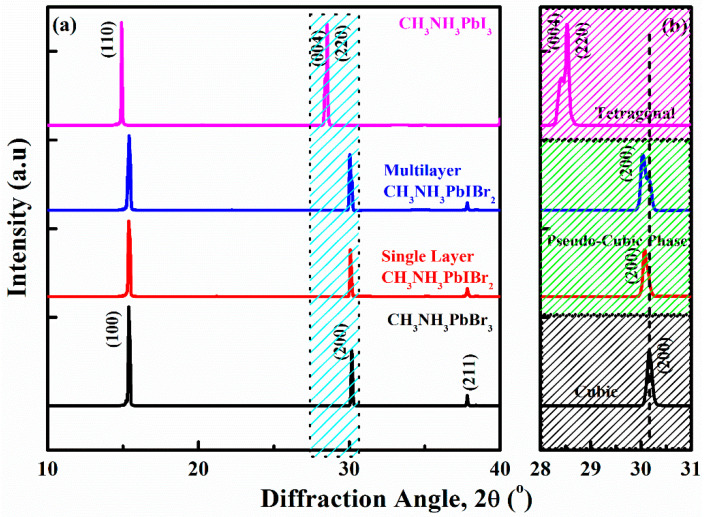
(**a**) XRD analysis of CH_3_NH_3_PbI_3_ thinfilm (as a reference at top), CH_3_NH_3_PbBr_3_ thinfilm (as a reference at bottom), single-layer and multilayered CH_3_NH_3_PbIBr_2_ thinfilms (at center). (**b**) Amplified XRD image from 2θ = 28–31° to observe the pseudo-cubic phase for single-layer and multilayered CH_3_NH_3_PbIBr_2_ thinfilms.

XRD analysis for CH_3_NH_3_PbI_3_ thinfilm, (top), CH_3_NH_3_PbIBr_2_ (single-layer thinfilm), CH_3_NH_3_PbIBr_2_ (multilayer thinfilm) and CH_3_NH_3_PbBr_3_ thinfilm (bottom) are demonstrated in Figure 1a, measured at room temperature. All the samples have maintained the pure perovskite phases. In the XRD measurements of the perovskite thinfilms, sharp intensity peaks at 14.5°, and 28.5°, are associated with (110) and (220) diffractions of CH_3_NH_3_PbI_3_ (top) confirming the perovskite structure for halide material. Two hump-like peaks located at 28.2° and 28.5°, are assigned to the (004) and (220) lattice planes for the tetragonal *I4/mcm* phase [9,18]. Meanwhile, CH_3_NH_3_PbBr_3_ thinfilm (bottom), CH_3_NH_3_PbIBr_2_ (single-layer thinfilm) and CH_3_NH_3_PbIBr_2_ (multilayer thinfilm) are crystalized and indexed in a cubic structure with a $Pm\bar{3}m$ space group symmetry. Sharp intensity peaks at 14.9°, 30.16° and 37.8° are associated with (100), (200) and (211) lattice planes of the cubic structure [19]. Herein, it is noticeable that for CH_3_NH_3_PbIBr_2_ (single-layer thinfilm) and CH_3_NH_3_PbIBr_2_ (multilayer thinfilm), all the intensity peaks display obvious shoulders, where diffraction peaks of both samples are symmetrical with the domination of the cubic phase. The tetragonal phase of the pure CH_3_NH_3_PbI_3_ thinfilm is basically a transition from the cubic phase by the slight rotation of PbI_6_ octahedra along the (001) axis on the (001) plane while retaining their corner-sharing connectivity; that is why the mix halides can be designated by a pseudo-cubic lattice [20]. Preferably, the perovskite materials possess cubic symmetry, but whenever they are transformed to pseudo-cubic or distorted cubic symmetry, either due to the grain size or to mixing the halides, this variation in the structure brings substantial variations in the properties of perovskite [21].

**Figure 2:**
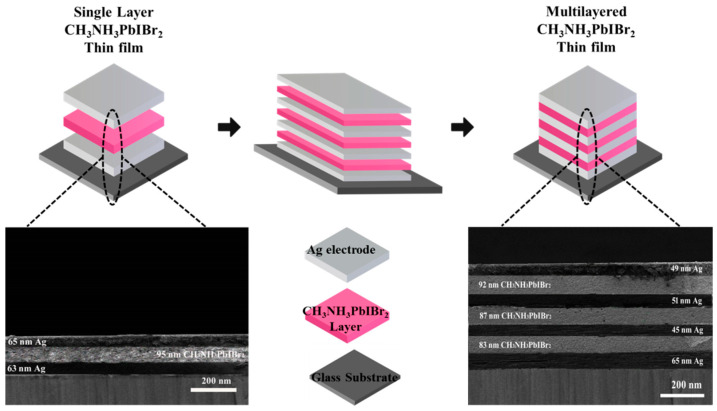
Schematic illustration of synthesized single-layer and multilayered CH_3_NH_3_PbIB_2_ thinfilms, along with the cross-sectional SEM images.

The schematic illustration of single-layer and multilayered growth of thinfilms is described in Figure 2, consisting of four alternating layers of Ag electrodes and three layers of CH_3_NH_3_PbIBr_2_ on the glass substrate. Adhesion to the substrate surface is an important requirement, especially in the outdoor environment, where the coating is performed, i.e., atmospheric agents and heavy cleaning treatments.

**Figure 3:**
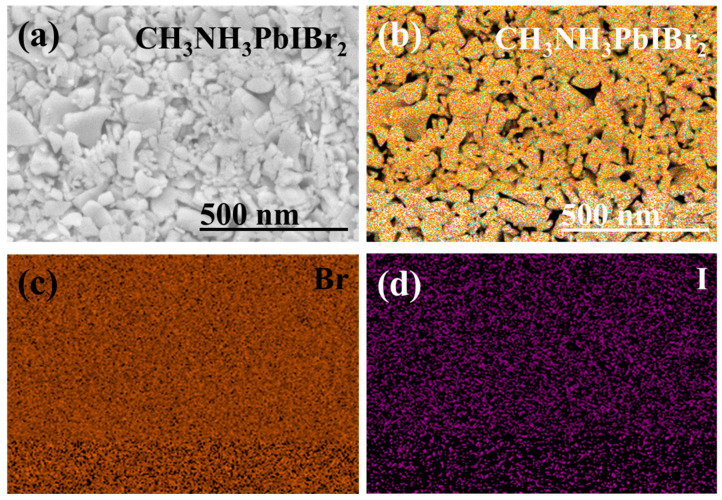
(**a**) Surface SEM analysis of multilayered top layer of CH_3_NH_3_PbIBr_2_ thinfilm, (**b**) colored FESEM elemental analysis multilayered CH_3_NH_3_PbIBr_2_ thinfilm, Dispersion of Br (**c**) and I (**d**).

Herein, the cross-section SEM images of single-layer CH_3_NH_3_PbIBr_2_ thinfilm and multilayered CH_3_NH_3_PbIBr_2_ thinfilm are illustrated. The overall single-layer thinfilm thickness is ~340 nm, consisting of a pseudo-cubic CH_3_NH_3_PbIBr_2_ layer of ~95 nm, which is superimposed between the Ag (bottom 63 nm and top 65 nm electrodes) layers. Similarly, the thickness of the multilayered CH_3_NH_3_PbIBr_2_ thin film is ~585 nm with the average same thickness size (~88 nm) of the perovskite layers. For the elemental analysis and the elemental distribution of the dual halide (Br, I), constituents of the multilayer CH_3_NH_3_PbIBr_2_ thinfilm surface FE-SEM analysis are taken and illustrated in Figure 3. Figure 3a is the SEM surface image of CH_3_NH_3_PbIBr_2_ layer; the layer shows a loose and porous structure. Two dimensional elemental mappings of the surface states that Br and I elements possess the higher degree of dispersion and uniformity throughout the surface (Figure 3b–d).

**Figure 4:**
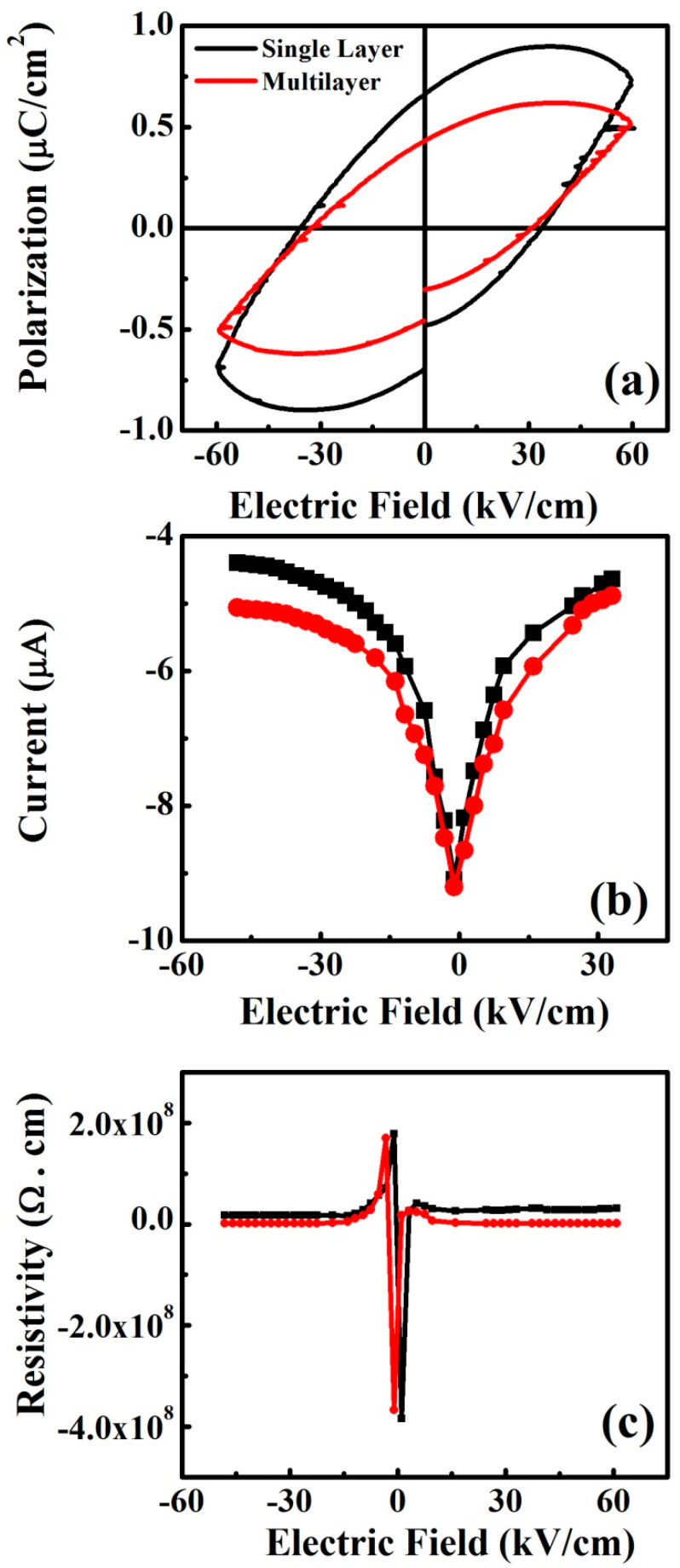
(**a**) Ferroelectric (*P-E*) loops for single-layer and multilayered CH_3_NH_3_PbIBr_2_ thinfilms, (**b**) current versus applied electric field for single-layer and multilayered CH_3_NH_3_PbIBr_2_ thinfilms, (**c**) resistivity versus applied electric field single-layer and multilayered CH_3_NH_3_PbIBr_2_ thinfilms.

Ferroelectric (*P-E*) loops of Ag/CH_3_NH_3_PbIBr_2_/Ag, single-layer and multilayer thinfilms grown on glass substrate are presented in Figure 4a, measured at 50 °C under the frequency of 10 Hz. Both the films have shown a leaky capacitor characteristic, which might be due to the presence of defects and high porosity (discussed in SEM analysis) in thinfilms which create percolation paths for the current. It is reported that the ferroelectric behavior in the tetragonal phased CH_3_NH_3_PbI_3_ propagates due to the ionic polarization by the off-center shift of Pb in the PbI_6_ octahedral [22], which plays a vital role to establish the hysteresis in the dual halide peovskite materials, i.e., CH_3_NH_3_PbIBr_2_. Herein, weak ferroelectric performance is observed in the Ag/CH_3_NH_3_PbIBr_2_/Ag single-layer and multilayer thinfilms, but these are improved results as compared to previous reports. Leakage current plays a vital role on the ferroelectric properties of the material. Figure 4b presents the leakage current versus electric field plots of CH_3_NH_3_PbIBr_2_ single phase and multiphase thinfilms, measured at 50 °C. A leakage current value of ~4.83 × 10^−6^ A has been detected for the single-layer Ag/CH_3_NH_3_PbIBr_2_/Ag thinfilm at the electric field of ~50 kV/cm. The value increased to 5.06 × 10^−6^ A for the multilayered thinfilm at the same applied electric field. Moreover, in multilayer films, the particle size increased due to reduced lattice mismatching. This increment in particle size reduces the grain boundaries that produce better electron mobility in the multilayer thinfilms. The number of extra Ag electrode layers in the multilayered thinfilms is another reason for this increment in the leakage current. In addition, both of them exhibited ohmic contact with Ag as there was no rectification on the contact region. Hence, they did not show diode-like behavior. Figure 4c is the resistivity versus electric field plots of CH_3_NH_3_PbIBr_2_ single phase and multiphase thinfilms. Single layer Ag/CH_3_NH_3_PbIBr_2_/Ag thinfilm presented a high resistivity of 1.87 × 10^6^ Ω.cm at the applied electric field of ~50 kV/cm, and resistivity reduced for the multilayered thinfilm to 1.60 × 10^6^ Ω.cm, as was expected after following the trends of ferroelectric and leakage current plots.

**Figure 5:**
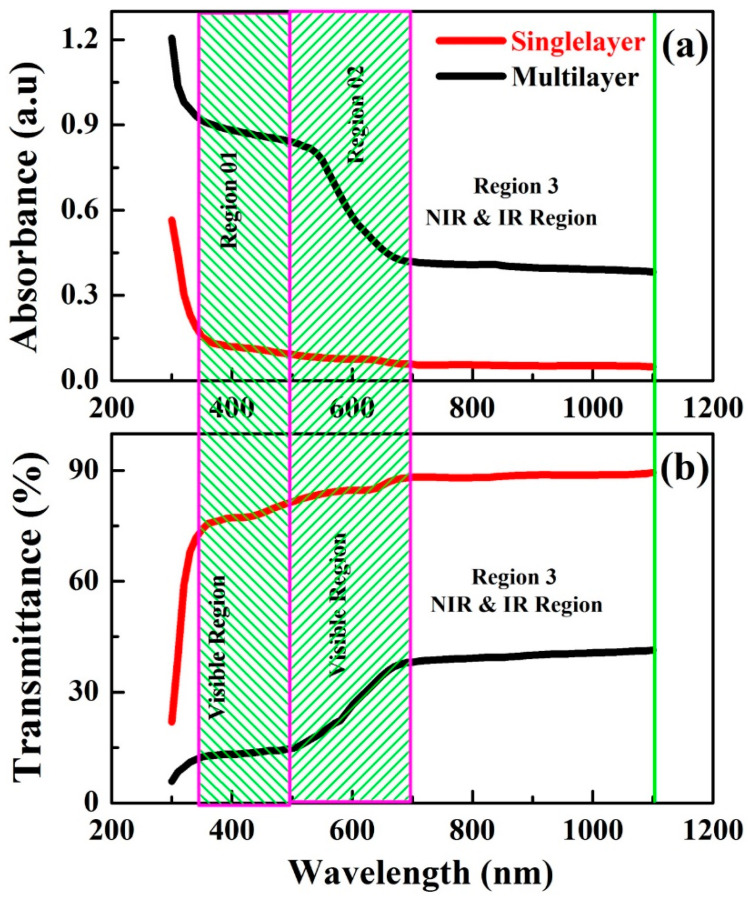
(**a**) Absorbance, (**b**) transmittance, spectra for single-layer and multilayered CH_3_NH_3_PbIBr_2_ thinfilms.

Figure 5a shows the absorbance spectra for the wavelength range of 300–1100 nm measured at room temperature. Samples showed two different trends for the absorbance spectra. Both films showed a decreasing trend of absorbance in the visible region (350–700 nm), whereas the absorbance becomes lower for the infrared/near infrared (IR/NIR) region; this decrease is due to lower production of excitation at the bandgap by the energy of both samples. Furthermore, multilayered perovskite film showed the maximum absorbance in the wavelength range of 300–450 nm and then showed it reduced for the wavelength range of 450–650 nm. Hence, it shows that for region 1, more absorbance is achieved as compared to region 2, and later it decreased further with the increase in wavelength 650–1100 nm in the IR/NIR region 3. The absorbance behavior of multilayered perovskite thinfilm produces the maximum quantum efficiency as compared to single-layer. Figure 5b shows the transmittance spectra for the wavelength range 300–1100 nm measured at room temperature. In IR/NIR region (650 nm to 1050 nm), the curve obtained for single-layered thinfilm depicted 90% of the maximum transmittance of light. However, in the visible region of light, the sample shows 80–85% transmittance with sharp absorbance. In contrast, the multilayered (CH_3_NH_3_PbIBr_2_) thinfilm shows 45% of light transmittance in the IR/NIR region, which varies from 650 nm–1050 nm. On the other hand, in the visible region starting from 350 nm to 650 nm, it transmits 15–20% of the light. Hence, transmittance of light in both samples increases with increasing wavelength and decreasing energy, because the energy of incoming photons does not produce excitations across the band gap. Single-layered perovskite thinfilm exhibits maximum transmittance as compared to multilayered thinfilm because of its minimum thickness.

**Figure 6:**
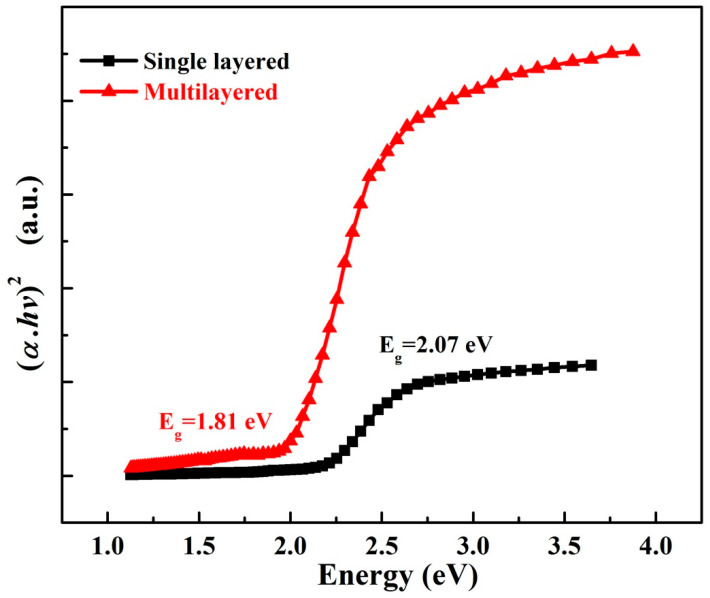
Tuac plot for single-layer and multilayered CH_3_NH_3_PbIBr_2_ thinfilms.

Figure 6 is the plot measured by Tauc plot method which is widely implemented for the determination of the bandgap value of the materials. Herein, bandgaps of single-layered and multilayered CH_3_NH_3_PbIBr_2_ thinfilms are calculated.

For the band gap estimation the Tauc plot method is used, and the band gap for single-layered thin films is 2.07 eV, which is consistent with the previous report [23]. Meanwhile, the bandgap of CH_3_NH_3_PbIBr_2_ multilayered thinfilm is 1.81 eV. It shows that the bandgap is dependent on the thickness of the thinfilms. Graphically, photon energy (*hv*) on the *x*-axis and the other quantity (*αhν*)^1/2^ on the y axis can be written as:

(1) $${\left(\alpha .hv\right)}^{\frac{1}{\gamma }}=B\left(hv-E_{g}\right)$$

where ν is the photon’s frequency, $E_{g}$ is the bandgap energy and B is a constant. The factor γ relies on the nature of transitions of electrons with values 1/2, 3/2 or 3 for direct-allowed, indirect-allowed, direct-forbidden and indirect-forbidden transitions [24]. A graph is plotted for direct-allowed transitions.

**Figure 7:**
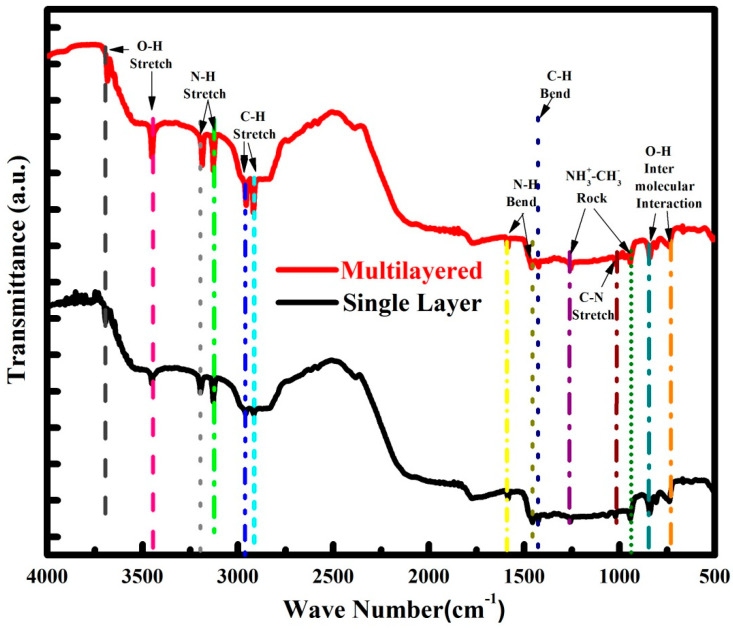
FTIR spectra for single-layer and multilayer CH_3_NH_3_PbIBr_2_ thinfilms.

In Figure 7, FTIR spectroscopy of single-layer CH_3_NH_3_PbIBr_2_ and multilayer CH_3_NH_3_PbIBr_2_ thinfilms are presented for the wavenumber range 500–4000 cm^−1^. Both films depicted similar behavior. FTIR is employed to observe the vibrational properties of the synthesized material. The band between 3682 cm^−1^ and 3452 cm^−1^ belongs to O-H stretching which may come from moisture in the films [25]. Peaks at 3186 cm^−1^ and 3126 cm^−1^ from N-H asymmetric stretching and N-H symmetric stretching, respectively. Peaks from 2956 cm^−1^ and 2918 cm^−1^ appear due to C-H asymmetric stretching and C-H symmetric stretching, respectively [26]. Similarly, the less intensity peaks at the wavelengths 1585 cm^−1^, 1460 cm^−1^ and 1423 cm^−1^ are from asymmetric N-H bending, symmetric N-H bending and C-H bending, respectively [27]. Less intense peaks at 1255 cm^−1^ and 943 cm^−1^ belong to CH_3_-NH^3+^ rocking [28].

**Figure 8:**
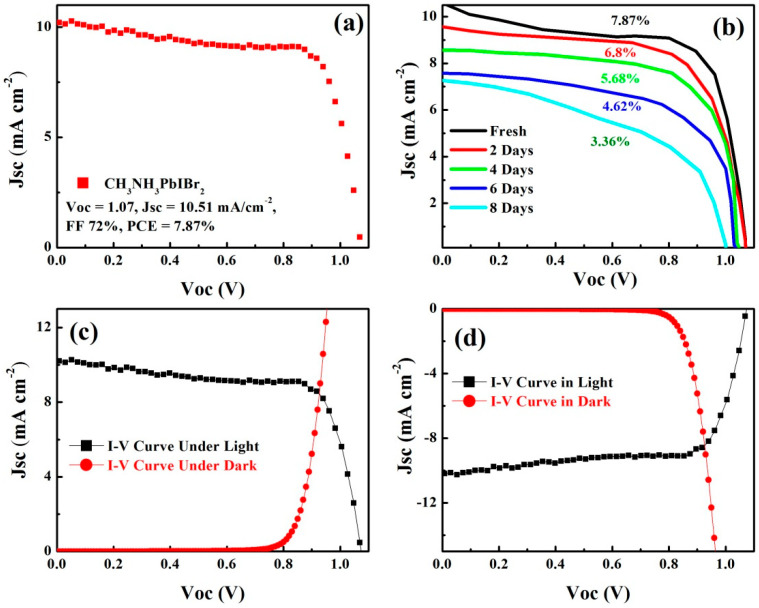
(**a**) Characteristic current–voltage (J-V) curve for photovoltaic cell based on CH_3_NH_3_PbIBr_2_ layer and its PV performance measured under AM 1.5G condition, (**b**) the stability (J-V curves) of perovskite solar cell based on CH_3_NH_3_PbIBr_2_ absorber material for 8 days, (**c**,**d**) the J-V curve behaviors in light and dark.

Lastly the photovoltaic performance of fabricated dual halide perovskite material CH_3_NH_3_PbIBr_2_ is observed as an absorber layer in the perovskite solar cells (Figure 8). For this purpose, an inverted device structure is used, which is based on ITO/Poly (triaryl amine) (PTAA) (10 nm)/CH_3_NH_3_PbIBr_2_ (perovskite layer)/(6,6)-Phenyl-C61-butyric acid methyl ester (PCBM) (50 nm)/Al (100 nm). Here, PTAA and PCBM are employed as hole- and electron-transport layers, respectively. Characteristic current–voltage (J-V) curve for perovskite solar cells based on CH_3_NH_3_PbIBr_2_ is displayed in Figure 8, and PV performance measured under AM 1.5G condition. The highest power conversion efficiency (PCE) achieved with this absorber material was 7.87% along with a decent open circuit voltage of 1.07 V, short circuit current density (Jsc) of 10.51 mA cm^−2^ and a fill factor (FF) of 72%. The stability (J-V) of a perovskite solar cell based on a CH_3_NH_3_PbIBr_2_ absorber material for 8 days is measured under a controlled and ambient environment at room temperature with relative humidity of 45%, as shown in Figure 8b. The device decomposed 5% and 20% under a controlled and ambient atmosphere, respectively. Wide band gap-based perovskite absorber materials are an emerging area of research in perovskite photovoltaic. In this study, we used an inverted planar structure of perovskite solar cells, which is lagging behind regular planar and mesoporous structures in terms of efficiency. The main advantages of this inverted structure are the blockage of ion migration and better stability as compared to other structures. As per our knowledge, 7.87% efficiency is still the highest in an inverted planar with CH_3_NH_3_PbIBr_2_ structure and also comparable with a regular planar structure in PCE. Figure 8c,d are the J-V behaviors of a solar cell based on a CH_3_NH_3_PbIBr_2_ absorber material under the light and dark environment.

Here, a final comparison is made bwteen the perovskite solar cell based on a CH_3_NH_3_PbIBr_2_ absorber material (this work), shown in Table 1, and the previously reported different materials-based solar cells. The reported material showed better performance than all other mentioned materials.

## 4. Conclusions

In this research study, single- and multilayered semiconductor CH_3_NH_3_PbIBr_2_ thinfilms were synthesized; XRD analysis confirmed the single-phase perovskite structure of the films with the existence of pseudo-cubic phase (200) at 30.16°. Leaky ferroelectric behavior was depicted by both thinfilms, while multilayered thinfilm showed a leakage current of ~5.06 × 10^−6^ A and resistivity of ~1.60 × 10^6^ Ω.cm for the applied electric field of 50 kV/cm. Optical comparison showed that the multilayered perovskite thinfilms produced the maximum quantum efficiency as compared to single-layer. A Tuac plot reveals the band gap calculation of 2.07 eV and 1.81 eV for single- and multilayered films, respectively. Photovoltaic performance of the material as an absorber layer in the photovoltaic cell was observed with the PCE of 7.87% and FF of 72%. The results illustrate the abilities of the engineered material to be utilized in optoelectronic and photovoltaic devices.

## Figures and Tables

**Table 1:** Performance of solar cells.

No	Materials	*J_SC_* (mA/cm^2^)	*V_OC_* V	PCE %	Ref.
1	HNO_3_/PFSA/Gr/oxide/n-Si	32.46	0.521	10.44	[29]
2	MoS_2_ (monolayer CVD)/*p*-Si	22.36	0.41	5.23	[30]
3	Bilayer graphene/MoS_2_/*n*-Si	21.4	0.51	5.98	[31]
4	Trilayer graphene/MoS_2_/*n*-Si solar cell	33.4	0.56	11.1	[31]
5	MoS_2_/*h*-BN/GaAs (AuCl_3_ doped)	20.8	0.64	7.15	[32]
6	MoS_2_/*h*-BN/GaAs (AuCl_3_ doped) *V_gate_* = −1.0 V	21.1	0.76	9.03	[32]
7	Glass/FTO/compact-TiO_2_/mesoporous-TiO_2_/CH_3_NH_3_PbI_3_/MoS_2_/Spiro-OMeTAD/Au solar cells	21.5	0.93	13.3	[33]
8	ITO/Poly (triaryl amine) (PTAA)/CH_3_NH_3_PbIBr_2_/(6,6)-Phenyl-C61-butyric acid methyl ester (PCBM)/Al	10.51	1.07	7.87	This work

## Data Availability

Data sharing is not applicable to this article.
